# Clinical Features of COVID-19 Patients with Different Outcomes in Wuhan: A Retrospective Observational Study

**DOI:** 10.1155/2020/2138387

**Published:** 2020-10-05

**Authors:** Zhen Wang, Di Ye, Menglong Wang, Mengmeng Zhao, Dan Li, Jing Ye, Jianfang Liu, Yao Xu, Jishou Zhang, Wei Pan, Menglin Liu, Zhen Luo, Jun Wan

**Affiliations:** ^1^Department of Cardiology, Renmin Hospital of Wuhan University, Wuhan 430060, China; ^2^Cardiovascular Research Institute, Wuhan University, Wuhan 430060, China; ^3^Hubei Key Laboratory of Cardiology, Wuhan 430060, China; ^4^Department of Pediatrics, Renmin Hospital of Wuhan University, Wuhan, China; ^5^Department of Emergency, Renmin Hospital of Wuhan University, Wuhan, China

## Abstract

Coronavirus disease 2019 (COVID-19) has caused considerable morbidity and mortality worldwide since December 2019. This retrospective study determined the characteristics and prognostic factors of COVID-19 patients, focusing on inpatients who died or were discharged between 30 December 2019 and 29 February 2020 at Renmin Hospital of Wuhan University. Patients' medical histories, comorbidities, symptoms, signs, laboratory findings, computed tomography (CT) findings, and clinical management were recorded. All 293 patients were divided into the nonsurviving (*n* = 116) and surviving (*n* = 177) groups. The median age was older in the nonsurviving group than in the surviving group; most patients were older than 65 years in the nonsurviving group. The incidence rates of lymphopenia, neutrophilia, and leukocytosis were significantly higher in the nonsurviving group than in the surviving group. More patients in the nonsurviving group had increased levels of nonspecific infection markers, abnormal liver and kidney function, cardiac injury, and blood coagulation abnormalities on admission. Immune and inflammatory responses were more severely disturbed in the nonsurviving group than in the surviving group. The incidence rates of complications during hospitalization were higher in the nonsurviving group than in the surviving group. Cox regression results also showed that older age, symptoms of dyspnea, comorbidities, and complications were all predictors of death. Close monitoring and timely treatment are needed for high-risk COVID-19 patients.

## 1. Introduction

In late December 2019, cases of pneumonia of an unknown cause emerged in Wuhan and spread to most parts of China [1–3]. The gene sequence of the virus obtained from these patients showed that the new virus is a member of the *Coronaviridae* family; this new virus was subsequently named severe acute respiratory syndrome coronavirus 2 (SARS-CoV-2) [4, 5]. On February 12, 2020, the disease caused by SARS-CoV-2 was named coronavirus disease 2019 (COVID-19) [6].

As of July 15, 2020, the National Health Commission (NHC) of China had confirmed a total of 85677 cases of COVID-19 in mainland China, including 4649 deaths and 80407 recovered patients. Although China has gradually controlled the spread of COVID-19, many countries in the world are experiencing outbreaks, and the numbers of patients in those countries are sharply increasing, including in the United States of America, India, Brazil, and Russia. Thus, governments must work together to combat the COVID-19 epidemic.

The clinical spectrum of patients with COVID-19 appears to be wide, encompassing asymptomatic infection, mild upper respiratory tract illness, and severe viral pneumonia with respiratory failure and even death [7–10]. Although some case series have been published, knowledge about COVID-19 is still incomplete. The present study aims at exploring the clinical characteristics of patients with different outcomes (death or discharge) and prognostic factors, which might provide evidence for risk stratification and help to improve clinical practices and reduce fatality.

## 2. Methods

### 2.1. Data Source

We conducted a retrospective study focusing on inpatients who died or were discharged between 30 December 2019 and 29 February 2020 at Renmin Hospital of Wuhan University. All patients were diagnosed with COVID-19 according to the “Diagnosis and treatment of novel coronavirus pneumonia” formulated by the NHC of China. The criteria for discharge were the absence of fever for at least 3 days, substantial improvement in both lungs on chest computed tomography (CT), the clinical remission of respiratory symptoms, and two throat-swab samples that tested negative for SARS-CoV-2 RNA and were obtained at least 24 hours apart. According to prognosis, all 293 patients were divided into the nonsurviving group (*n* = 116) and the surviving group (*n* = 177). This study was approved by the Ethics Commission of Renmin Hospital of Wuhan University.

### 2.2. Data Collection

We collected data on sex, age, comorbidity (hypertension, diabetes, cerebrovascular disease, coronary heart disease, COPD, chronic renal disease, chronic liver disease, and malignancy), symptoms from onset to hospital admission (fever, respiratory symptoms, catarrhal symptoms, digestive symptoms, and neuromuscular symptoms), vital signs (heart rate, blood pressure, respiratory rate, and finger oxygen saturation), laboratory values on admission (complete blood count, blood chemistry tests, liver and kidney function, cardiac and skeletal muscle injury, arterial blood gas analysis, electrolytes, coagulation function, humoral immunity, and cellular immunity), treatment (oxygen therapy, antiviral treatment, antibiotic treatment, glucocorticoids, immunoglobulin therapy, and antifungal treatment), and complications (shock, acute cardiac injury, acute renal injury, and acute liver injury).

### 2.3. Statistical Analysis

Continuous variables are presented as either the means ± standard deviations (SDs) or the medians (with interquartile ranges). For categorical variables, we calculated the frequency rates and percentages of patients in each category. Continuous variables were compared using independent group *t* tests when the data were normally distributed; otherwise, the Mann–Whitney test was used. Proportions for categorical variables were compared using the *χ*^2^ test, although Fisher's exact test was used when the data were limited. Cox proportional hazard regression models were used to determine the prognostic factors, and the hazards ratio (HR) and 95% confidence interval (95% CI) were reported. Statistical analyses were performed using SPSS 21.0 and GraphPad Prism 7.

## 3. Results

### 3.1. Presenting Characteristics

We included 293 inpatients in this study: 116 patients died during hospitalization, and 177 patients were discharged. The results showed that the median age of the nonsurviving group was higher than that of the surviving group (72.5 [63.5, 81] vs. 51 [34, 64]). Regarding the initial symptoms, the nonsurviving group had more patients with respiratory symptoms at admission (77.6% vs. 60.5%), while there was no difference in fever, catarrhal symptoms, neuromuscular symptoms, or digestive symptoms between the two groups. In addition, the temperature (36.8 [36.5, 37.2] vs. 36.6 [36.4, 36.9]), heart rate (86 [77, 102] vs. 80 [76, 90]), and respiratory rate (20 [19, 27] vs. 20 [18, 20]) on admission and the highest temperature (38.5 [37.5, 39.1] vs. 37.3 [37, 37.8]) during hospitalization were higher in the nonsurviving group than in the surviving group. The finger oxygen saturation level (92 [84, 97] vs. 98 [96, 99]) was lower in the nonsurviving group than in the surviving group, while blood pressure was not significantly different between the two groups. Moreover, the nonsurviving group had more patients with comorbidities, including hypertension (56.9% vs. 14.7%), diabetes (19.8% vs. 7.9%), cerebrovascular disease (16.3% vs. 1.7%), coronary heart disease (12.1% vs. 4.0%), and chronic renal disease (7.8% vs. 1.1%). The clinical data for all patients are listed in Table 1.

### 3.2. Results of Laboratory Tests and CT Parameters

The first results of the laboratory tests and CT parameters after admission were analyzed. The routine blood test results showed that the white blood cell count (7.95 [5.42, 12.1] vs. 5.03 [3.98, 6.35]) and neutrophil count (6.67 [4.22, 10.6] vs. 2.19 [3.98, 6.35]) were higher and the lymphocyte count (0.62 [0.41, 0.87] vs. 1.34 [0.96, 1.63]) and platelet count (171.5 [117.5, 214.3] vs. 214 [168, 273]) were lower in the nonsurviving group than in the surviving group, while the red blood cell count was not significantly different between the two groups. In addition, the aspartate aminotransferase (39 [28, 58.8] vs. 25 [20, 34]), total bilirubin (12.8 [8.6, 19.6] vs. 10.7 [8, 14.3]), direct bilirubin (5.3 [3.4, 8.57] vs. 3.55 [2.78, 4.75]), creatinine (72.5 [55, 99.5] vs. 57.5 [48.8, 68.3]), blood urea nitrogen (7.62 [5.42, 13.2] vs. 4.1 [3.44, 5.0]), creatine kinase (97.5 [57.8, 236] vs. 57 [39, 88.5]), creatine kinase-myocardial isoenzyme (2.38 [1.3, 3.98] vs. 0.86 [0.57, 1.21]), lactate dehydrogenase (478 [342, 623] vs. 222.5 [188, 283.8]), myoglobin (106.1 [68.6, 334.8] vs. 34.6 [23.5, 52.5]), hypersensitive troponin I (0.044 [0.015, 0.131] vs. 0.006 [0.006, 0.006]), and D-dimer (2.82 [0.91, 12.9] vs. 0.66 [0.31, 1.36]) levels and activated partial thromboplastin time (29.5 [27.7, 32.91] vs. 27.95 [25.4, 30.9]) were higher, but the total protein level (58.7 [54.9, 62.3] vs. 62.4 [59, 65.9]), albumin level (33.3 [30.9, 36.4] vs. 38.5 [35.3, 41.7]), arterial oxygen saturation level (89 [80, 95] vs. 97 [95, 98]), arterial partial pressure of oxygen (57 [45, 75] vs. 87 [74, 111]), arterial partial pressure of carbon dioxide (37 [33, 43] vs. 42 [38, 45.3]), lactic acid level (23.6 [20.7, 26.7] vs. 26.1 [24.2, 27.7]), and prothrombin time activity (74.9 [64.1, 85.4] vs. 84.7 [77.4, 96.9]) were lower in the nonsurviving group than in the surviving group.

The tests of the inflammatory and immune responses showed that the levels of C-reactive protein (86.1 [54, 168.9] vs. 11.6 [5, 41.4]), high-sensitivity C-reactive protein (5 [5] vs. 5 [1.78, 5]), procalcitonin (0.18 [0.1, 0.55] vs. 0.04 [0.03, 0.07]), immunoglobulin A (2.55 [1.9, 3.63] vs. 2.19 [1.72, 2.73]), and immunoglobulin E (67.1 [31.4, 188] vs. 37 [18.3, 108]); the CD16+56 T cell proportion (19.2 [10.3, 29.3]% vs. 11.6 [6.43, 17.0]%); and the CD19 T cell proportion (19.1 [11.7, 27.9]% vs. 14.3 [11.4, 19.2]%) were higher in the nonsurviving group than that in the surviving group; however, the CD16+56 T cell count (97 [41.5, 168.5] vs. 119.5 [77.5, 180.3]), CD19 T cell count (91 [55.5, 146.5] vs. 159 [109.8, 226]), CD3 T cell proportion (54.2 [43.3, 65.0]% vs. 69.8 [61.0, 75.2]%), CD3 T cell count (272 [185.5, 405] vs. 781 [582.4, 1023.8]), CD4 T cell proportion (32.1 [26.7, 41.6]% vs. 41.8 [36.3, 46.9]%), CD4 T cell count (168 [106.5, 269] vs. 486 [357.8, 651.5]), CD8 T cell proportion (16.0 [10.9, 24.4]% vs. 24.0 [18.8, 29.6]%), and CD8 T cell count (73 [46.5, 131.5] vs. 263 [166.8, 396]) were lower in the nonsurviving group than in the surviving group.

The results of CT imaging also showed that there were no significant differences in pneumonia, affected area, air bronchus signs, or consolidation between the two groups. The laboratory tests and CT parameters for all patients are listed in Tables 2–4.

### 3.3. Complications and Treatment

The results showed that the nonsurviving group had more patients with complications (68.1% vs. 6.8%), including shock (44.0% vs. 2.8%), acute cardiac injury (57.8% vs. 2.8%), acute renal injury (17.2% vs. 0.6%), and acute liver injury (10.3% vs. 4.0%). In terms of treatment, more patients received ICU care (31.9% vs. 2.8%) in the nonsurviving group than in the surviving group. Regarding medication, more patients in the nonsurviving group received oxygen therapy (76.7% vs. 46.9%), glucocorticoids (68.1% vs. 33.9%), immunoglobulin treatment (61.2% vs. 40.1%), and antifungal treatment (6.9% vs. 0.6%). The complications and treatments for all patients are listed in Table 5.

### 3.4. Prognostic Factors of the COVID-19 Patients

**Figure 1:**
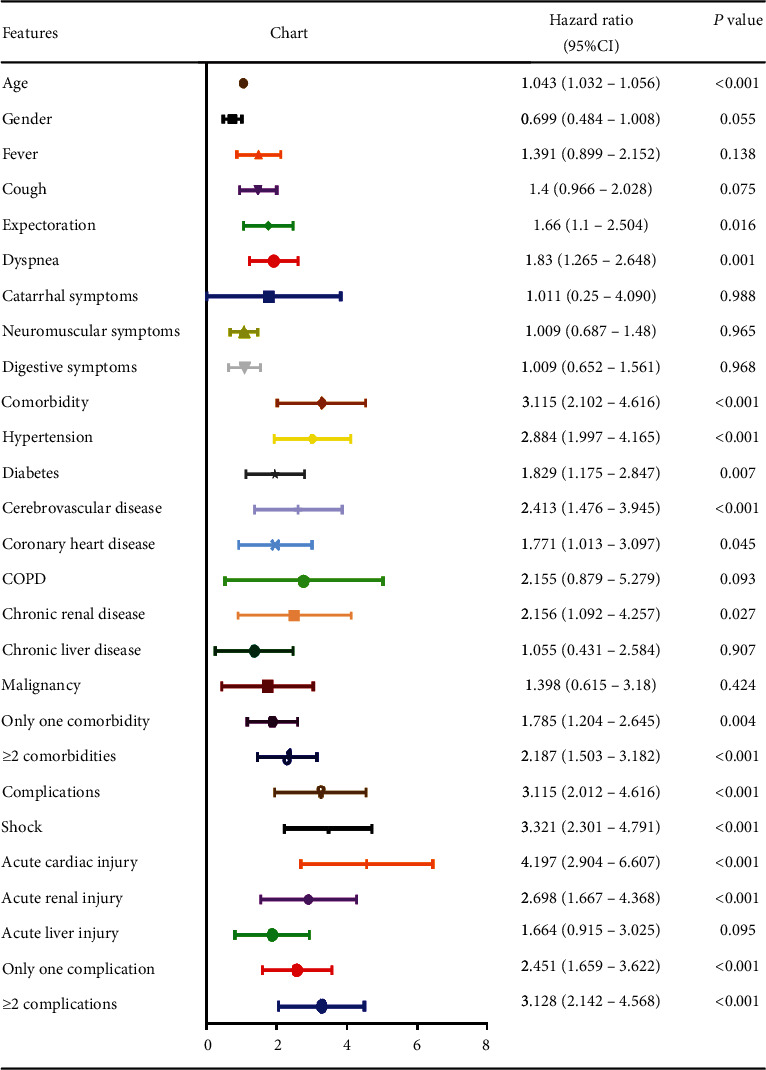
Cox regression analysis of the risk factors associated with mortality in patients with COVID-19.

Cox regression was used to analyze the prognostic factors of the COVID-19 patients. The results showed older age (HR 1.043, 95% CI 1.032-1.056, *P* < 0.001), symptoms of dyspnea (HR 1.83, 95% CI 1.265-2.648, *P* = 0.001), comorbidities including hypertension (HR 2.884, 95% CI 1.997-4.165, *P* < 0.001), diabetes (HR 1.829, 95% CI 1.175-2.847, *P* = 0.007), cerebrovascular disease (HR 2.413, 95% CI 1.476-3.945, *P* < 0.001), coronary heart disease (HR 1.771, 95% CI 1.103-3.097, *P* = 0.045), and chronic renal disease (HR 2.156, 95% CI 1.092-4.257, *P* = 0.027), and complications including shock (HR 3.321, 95% CI 2.301-4.791, *P* < 0.001), acute cardiac injury (HR 4.197, 95% CI 2.904-6.607, *P* < 0.001), and acute kidney injury (HR 2.698, 95% CI 1.667-4.368, *P* < 0.001) were all predictors of death. The Cox regression analysis are listed in Figure 1.

## 4. Discussion

In this study, we retrospectively analyzed the clinical characteristics of patients with different outcomes (death or discharge) and described the clinical course of symptoms, laboratory findings, and treatment process during hospitalization. The results showed that the median age in the nonsurviving group was older than that in the surviving group, and a majority of patients were older than 65 years in the nonsurviving group. The incidences of lymphopenia, neutrophilia, and leukocytosis in the nonsurviving group were significantly higher than those in the surviving group. More patients in the nonsurviving group had increased nonspecific markers of infection, abnormal liver and kidney function, cardiac injury, and blood coagulation abnormalities on admission. The disturbances of the immune and inflammatory responses were more severe in the nonsurviving group than in the surviving group. In addition, the incidences of complications during hospitalization were higher in the nonsurviving group than in the surviving group. Cox regression results also showed older age, symptoms of dyspnea, comorbidities, and complications were all predictors of death.

Huang et al. first reported the clinical characteristics of 41 patients with COVID-19 in Wuhan city. The results showed that patients' clinical manifestations included fever, cough, dyspnea, myalgia, fatigue, lymphopenia, and radiographic evidence of pneumonia [11]. In this cohort, we found that most patients presented with fever, dry cough, dyspnea, and bilateral ground-glass opacities on chest CT scans. However, no difference in fever, catarrhal symptoms, neuromuscular symptoms, digestive symptoms, or chest CT scan findings was found between the surviving and nonsurviving patients. The Cox regression results also suggested that expectoration and dyspnea, but not fever, cough, catarrhal symptoms, neuromuscular symptoms, and digestive symptoms, can be used as indicators of the prognosis of the disease. However, further studies are needed to test this conclusion.

A previous study reported findings from 99 patients with COVID-19, and the results suggested that SARS-CoV-2 infection in humans was more likely to affect older men with comorbidities and may result in severe respiratory diseases [12]. Zhao et al. also reported that older patients were more likely to develop critical illness with a significantly higher mortality rate [13]. Consistent with these studies, in this cohort, we observed a greater number of men than women, and the patients were older in the nonsurviving group than in the surviving group. Additionally, approximately half of the patients in the nonsurviving group had chronic underlying diseases, mainly cardiovascular and cerebrovascular diseases and diabetes; this is similar to the findings reported in patients infected with SARS­CoV and MERS­CoV [14–18]. The Cox regression results also suggest that older age and the presence of comorbidities, including hypertension, diabetes, cerebrovascular disease, coronary heart disease, and chronic renal disease, might be risk factors for a poor outcome as a result of the weaker immune function of these patients.

Some studies have suggested that SARS­CoV-2 virions spread through the respiratory mucosa and infect other cells, induce cytokine storms in the body, generate a series of immune responses, and cause changes in peripheral white blood cells and immune cells such as lymphocytes [12, 19]. In our cohort, lymphocytopenia occurred in approximately 86.6% of the patients in the nonsurviving group, which was a greater proportion than the 32.9% of patients in the surviving group. This result suggests that a substantial decrease in the total number of lymphocytes might be an important factor leading to the exacerbation of disease in patients and could act as a prognostic factor. In addition, we observed that the white blood cell count, neutrophil count, and blood urea nitrogen, creatinine, creatine kinase-myocardial isoenzyme, myoglobulin, troponin I, and D-dimer levels were higher in the nonsurviving group than in the surviving group. Neutrophilia may be associated with secondary infection, while coagulation activation could be related to sustained inflammatory responses. Acute heart and kidney injury could be related to direct effects of the virus or possibly hypoxia [20, 21].

SARS-CoV-2 infection was initially thought to mainly cause severe pneumonia and respiratory distress [22, 23]. However, increasing numbers of clinical studies have shown that a large number of patients with COVID-19 also present with cardiac, renal, and liver injuries [24–27]. Shi et al. also showed that cardiac injury is a common condition in patients with COVID-19 and is associated with higher mortality [24]. Pei et al. reported that renal abnormalities occurred in most patients with COVID-19 and were associated with a higher risk of in-hospital mortality [25]. Similar to these reports, our study also showed that compared with the surviving group, the nonsurviving group had more patients with complications, including shock, acute cardiac injury, acute renal injury, and acute liver injury. The Cox regression results also suggest that shock, acute cardiac injury, and acute kidney injury may be risk factors for a poor outcome among COVID-19 patients. Thus, complications must be identified and treated as soon as possible to reduce mortality and improve the quality of life of patients.

Our study has several limitations. First, although we obtained data from 293 patients with different outcomes, the cohort was still relatively small. More patients need to be analyzed to provide a comprehensive and precise description of the spectrum of COVID-19. Second, our study reports primarily baseline results from patients upon hospital admission, and more longitudinal data regarding disease progression need to be collected and studied.

In summary, the potential risk factors of older age, symptoms of dyspnea, comorbidities including hypertension, diabetes, cerebrovascular disease, coronary heart disease, and chronic renal disease, and the occurrence of shock, acute cardiac injury, and acute kidney injury may help clinicians identify patients with a poor prognosis. Close monitoring and timely treatment should be performed for patients with COVID-19 who are at high risk of a poor outcome.

## Figures and Tables

**Table 1:** Baseline characteristics of COVID-19 patients.

	All (*n* = 293)	Nonsurviving (*n* = 116)	Surviving (*n* = 177)	*P* value
Male (*n*, %)	138 (47.1%)	65 (56.0%)	73 (41.2%)	0.013
Age, median (IQR), y	59.2 (42.8, 73.1)	72.5 (64.8, 80.9)	50.7 (34.2, 63.9)	<0.001
Age group, No. (%)				
<45 years	77 (26.3%)	3 (2.6%)	74 (41.8%)	
45-64 years	88 (30.0%)	26 (22.4%)	62 (35.0%)	
65-84 years	105 (35.8%)	68 (58.6%)	37 (20.9%)	
≥85 years	23 (7.8%)	19 (16.4%)	4 (2.3%)	
Initial symptoms, No. (%)				
Fever	209 (71.3%)	90 (77.6%)	119 (67.2%)	0.055
Respiratory system symptoms	197 (67.2%)	90 (77.6%)	107 (60.5%)	0.002
Sore throat	11 (3.8%)	1 (4.0%)	10 (3.9%)	0.035
Cough	150 (51.2%)	69 (59.5%)	81 (45.8%)	0.022
Expectoration	50 (17.1%)	31 (26.7%)	19 (10.7%)	<0.001
Chest tightness	63 (21.5%)	32 (27.6%)	31 (17.5%)	0.040
Chest pain	8 (2.7%)	1 (0.9%)	7 (4.0%)	0.112
Dyspnea	81 (27.6%)	48 (41.4%)	33 (18.6%)	<0.001
Catarrhal symptoms	5 (1.7%)	2 (1.7%)	3 (1.7%)	0.985
Neuromuscular symptoms	101 (34.5%)	51 (44.0%)	60 (33.9%)	0.799
Weakness	88 (30.0%)	38 (32.8%)	50 (28.2%)	0.410
Dizziness	13 (4.4%)	8 (6.9%)	5 (2.8%)	0.979
Headache	10 (3.4%)	3 (2.6%)	7 (4.0%)	0.528
Muscle ache	17 (5.8%)	6 (5.2%)	11 (6.2%)	0.709
Digestive symptoms	65 (22.2%)	26 (22.4%)	39 (22.0%)	0.939
Anorexia	45 (15.2%)	20 (17.2%)	25 (14.1%)	0.469
Nausea	6 (2.0%)	3 (2.6%)	3 (1.7%)	0.598
Vomiting	7 (2.4%)	4 (3.4%)	3 1.7%)	0.337
Abdominal pain	2 (0.7%)	1 (0.9%)	1 (0.6%)	0.763
Diarrhea	19 (6.5%)	3 (2.6%)	16 (9.0%)	0.028
Characteristics on admission				
Temperature, median (IQR), °C ^a^	36.7 (36.4, .37.0)	36.8 (36.5, 37.1)	36.6 (36.4, 36.9)	0.025
≥37.3°C (%)	39 (15.9%)	18 (21.2%)	21 (13%)	0.097
Heart rate, median (IQR), bpm ^b^	81 (76,95)	86 (78, 102)	80 (76, 90)	0.006
Systolic pressure, median (IQR), mmHg ^c^	126 (116,136)	130 (116, 144)	125 (117,132)	0.050
Diastolic pressure, median (IQR), mmHg ^d^	76 (70,83)	78 (71, 85)	75 (69, 82)	0.055
Respiratory rate, median (IQR), bpm ^e^	20 (18, 21)	20 (19, 27)	20 (18, 20)	<0.001
Finger oxygen saturation, median (IQR), % ^f^	97 (93.5-, 99)	92 (84,97)	98 (96, 99)	<0.001
Highest temperature during hospitalization, median (IQR), °C ^g^	37.5 (37, 38.4)	38.5 (37.5, 39.0)	37.3 (37, 37.8)	<0.001
≥37.3°C	153 (63.5%)	67 (82.7%)	86 (53.8%)	<0.001
Comorbidity, No. (%)	122 (41.6%)	80 (69.0%)	42 (23.7%)	<0.001
Hypertension	92 (31.4%)	66 (56.9%)	26 (14.7%)	<0.001
Diabetes	37 (12.6%)	23 (19.8%)	14 (7.9%)	0.003
Cerebrovascular disease	22 (7.5%)	19 (16.3%)	3 (1.7%)	<0.001
Coronary heart disease	21 (7.2%)	14 (12.1%)	7 (4.0%)	0.009
COPD	11 (3.8%)	7 (6.0%)	4 (2.3%)	0.097
Chronic renal disease	11 (3.8%)	9 (7.8%)	2 (1.1%)	0.004
Chronic liver disease	12 (4.1%)	5 (4.3%)	7 (4.0%)	0.881
Malignancy	11 (3.8%)	6 (5.2%)	5 (2.8%)	0.302
Only one comorbidity	59 (20.1%)	36 (31.0%)	23 (13.0%)	<0.001
≥2 comorbidities	63 (21.5%)	44 (37.9%)	19 (10.8%)	<0.001

The total number of patients with available data: ^a^*n* (death) = 85, *n* (survival) = 161; ^b^*n* (death) = 83, *n* (survival) = 161; ^c^*n* (death) = 73, *n* (survival) = 131; ^d^*n* (death) = 73, *n* (survival) = 131; ^e^*n* (death) = 83, *n* (survival) = 161; ^f^*n* (death) = 49, *n* (survival) = 112; ^g^*n* (death) = 81, *n* (survival) = 160.

**Table 2:** General laboratory findings of COVID-19 patients on admission to the hospital.

Median (IQR)	All (*n* = 293)	Nonsurviving (*n* = 116)	Surviving (*n* = 177)	*P* value
Routine blood parameters ^a^				
White blood cell count, ×109/L	5.58 (4.22, 7.76)	7.95 (5.42, 12.12)	5.03 (3.98, 6.35)	<0.001
<3.5, No. (%)	32 (11.2%)	9 (8.0%)	23 (13.3%)	0.107
>9.5, No. (%)	43 (15.1%)	39 (34.8%)	4 (2.3%)	<0.001
Neutrophil count, ×109/L	3.89 (2.50, 6.38)	6.67 (4.23, 10.59)	2.92 (2.19, 4.38)	<0.001
Lymphocyte count, ×109/L	0.99 (0.64, 1.44)	0.62 (0.41, 0.87)	1.34 (0.96, 1.63)	<0.001
<1.1, No. (%)	154 (54.0%)	97 (86.6%)	57 (32.9%)	<0.001
Platelet count, ×109/L	195 (148, 249)	171.5 (117.5, 214.3)	214 (168, 273)	<0.001
<125, No. (%)	40 (14.2%)	33 (29.5%)	7 (4.0%)	<0.001
>350, No. (%)	17 (6.0%)	1 (0.9%)	16 (9.2%)	0.004
Red blood cell count, ×1010/L	4.10 (3.69, 4.51)	4.03 (3.65, 4.48)	4.15 (3.72, 4.52)	0.148
Liver function ^b^				
Alanine aminotransferase, U/L	24 (16, 40)	24 (18, 40)	24 (16, 40)	0.511
>150, No. (%)	6 (2.1%)	2 (1.8%)	4 (2.3%)	0.778
Aspartate aminotransferase, U/L	29 (21, 43)	39 (28, 59)	25 (20, 34)	<0.001
>120, No. (%)	7 (2.5%)	4 (3.7%)	3 (1.7%)	0.315
Total bilirubin, *μ*mol/L ^c^	11.4 (8.3, 16.2)	12.8 (8.6, 19.6)	10.7 (8, 14.3)	0.001
Direct bilirubin, *μ*mol/L ^c^	4.1 (3, 5.88)	5.3 (3.4, 8.57)	3.55 (2.78, 4.75)	<0.001
Total protein, g/L ^c^	61.1 (57.2, 64.8)	58.7 (54.9, 62.3)	62.4 (59, 65.9)	<0.001
Albumin, g/L ^c^	36.5 (33.0, 39.8)	33.3 (30.9, 36.4)	38.5 (35.3, 41.7)	<0.001
Kidney function ^d^				
Creatinine, *μ*mol/L	61 (51, 76)	72.5 (55, 99.5)	57.5 (48.8, 68.3)	<0.001
Increase, No. (%)	55 (19.5%)	48 (43.6%)	7 (4.1%)	<0.001
Blood urea nitrogen, nmol/L	4.88 (3.8, 7.2)	7.62 (5.42, 13.22)	4.1 (3.44, 5.0)	<0.001
Uric acid, *μ*mol/L	257.5 (208, 351.5)	267 (182.3, 395)	254 (214.8, 323.3)	0.405
Increase, No. (%)	60 (21.3%)	38 (34.5%)	22 (12.7%)	<0.001
Estimated glomerular filtration rate, mL/min	98.9 (82.3, 112.5)	84.0 (52.5, 97.0)	103.9 (96.2, 116.9)	<0.001
≤90, No. (%)	90 (31.9%)	64 (58.2%)	26 (15.1%)	<0.001
Injury of cardiac and skeletal muscles				
Creatine kinase, U/L ^e^	68 (43, 112)	97.5 (57.8, 236)	57 (39, 88.5)	<0.001
>310, No. (%)	28 (9.8%)	23 (20.9%)	5 (3.0%)	<0.001
Creatine kinase-myocardial isoenzyme mb, ng/mL ^f^	1.19 (0.64, 2.56)	0.82 (0.56, 1.22)	0.86 (0.57, 1.21)	<0.001
>5, No. (%)	15 (6.9%)	15 (14.8%)	0 (0)	<0.001
Lactate dehydrogenase, U/L ^g^	275 (202, 427)	473.5 (341, 612)	222.5 (188, 283.3)	<0.001
>250, No. (%)	159 (57.4%)	95 (85.6%)	64 (38.6%)	<0.001
Myoglobin, *μ*g/L h	57.6 (30.8, 116.4)	106.1 (68.6, 334.8)	34.6 (23.4, 52.5)	<0.001
>110, No. (%)	58 (27.2%)	50 (49.5%)	8 (7.1%)	<0.001
Hypersensitive troponin I, ng/mL ^i^	0.007 (0.006, 0.046)	0.044 (0.015, 0.131)	0.044 (0.015, 0.131)	<0.001
>0.0796, No. (%)	36 (16.7%)	36 (35.0%)	0 (0%)	<0.001
Arterial blood gas analysis ^j^				
Blood pH	7.42 (7.36, 7.46)	7.42 (7.35, 7.46)	7.41 (7.38, 7.45)	0.782
<7.35, No. (%)	30 (17.8%)	23 (23.7%)	7 (9.7%)	0.019
>7.45, No. (%)	56 (33.1%)	37 (38.1%)	19 (26.3%)	0.108
Arterial oxygen saturation, %	94 (88, 97)	89 (80, 95)	97 (95, 98)	<0.001
<95%, No. (%)	89 (52.7%)	72 (74.2%)	17 (23.6%)	<0.001
Arterial partial pressure of oxygen, mmHg	73 (54, 93)	57 (45, 75)	87 (74, 111)	<0.001
<60 mmHg, No. (%)	59 (34.9%)	53 (54.6%)	6 (8.3%)	<0.001
Arterial partial pressure of carbon dioxide, mmHg	40 (35, 45)	37 (33, 43)	42 (38, 45.3)	<0.001
<35 mmHg, No. (%)	40 (23.6%)	35 (36.1%)	5 (6.9%)	<0.001
>45 mmHg, No. (%)	45 (26.6%)	20 (20.6%)	25 (34.7%)	0.064
Lactic acid, mmol/L ^k^	2.3 (1.7, 3.2)	2.4 (1.8,3.4)	2.2 (1.55, 2.9)	0.063
Electrolytes ^l^				
K+, mmol/L	3.89 (3.5, 4.21)	3.8 (3.4, 4.12)	3.91 (3.56, 4.25)	0.202
Na+, mmol/L	139 (137, 142)	139 (135, 141.6)	140 (137, 142)	0.022
Cl-, mmol/L ^m^	106 (102.9, 108.3)	105.9 (101.6, 109.5)	106 (103.4,107.7)	0.610
Coagulation function				
Prothrombin time activity, % ^n^	81.7 (72.6, 91.4)	74.9 (64.1, 85.4)	78.85 (68.4, 89.03)	<0.001
Activated partial thromboplastin time, s ^o^	28.9 (26.3, 31.5)	29.5 (27.7, 32.9)	27.95 (25.4, 30.78)	<0.001
D-dimer, *μ*mol/L ^o^	1.02 (0.48, 3.69)	2.82 (0.91, 12.9)	0.66 (0.31, 1.36)	<0.001

The total number of patients with available data: ^a^*n* (death) = 112, *n* (survival) = 173; ^b^*n* (death) = 110, *n* (survival) = 173; ^c^*n* (death) = 110, *n* (survival) = 172; ^d^*n* (death) = 110, *n* (survival) = 172; ^e^*n* (death) = 110, *s*; ^f^*n* (death) = 101, *n* (survival) = 117; ^g^*n* (death) = 111, *n* (survival) = 166; ^h^*n* (death) = 101, *n* (survival) = 112; ^i^*n* (death) = 103, *n* (survival) = 112; ^j^*n* (death) = 97, *n* (survival) = 72; ^k^*n* (death) = 93, *n* (survival) = 71; ^l^*n* (death) = 114, *n* (survival) = 172; ^m^*n* (death) = 110, *n* (survival) = 172; ^n^*n* (death) = 104, *n* (survival) = 137; ^o^*n* (death) = 104, *n* (survival) = 136.

**Table 3:** Inflammatory responses and immunoreactions of COVID-19 patients on admission to the hospital.

Median (IQR)	All (*n* = 293)	Nonsurviving (*n* = 116)	Surviving (*n* = 177)	*P* value
Nonspecific inflammation index				
C-reactive protein, mg/L ^a^	36.6 (7.6, 85.4)	86.1 (54, 168.9)	11.6 (5, 41.4)	<0.001
>10, No. (%)	186 (70.2%)	104 (97.2%)	82 (51.9%)	<0.001
High-sensitivity C-reactive protein, mg/L ^b^	5 (5, 5)	5 (5, 5)	5 (1.78, 5)	<0.001
>5, No. (%)	40 (15.3%)	22 (20.6%)	18 (11.7%)	0.050
Procalcitonin, ng/mL ^c^	0.07 (0.04, 0.18)	0.18 (0.1, 0.55)	0.04 (0.03, 0.07)	<0.001
Humoral immunity ^d^				
Complement 3, g/L	0.98 (0.83, 1.13)	0.93 (0.79, 1.06)	1.02 (0.85, 1.14)	0.017
Complement 4, g/L	0.25 (0.19, 0.32)	0.24 (0.17, 0.32)	0.25 (0.2, 0.31)	0.337
Immunoglobulin A, g/L	2.55 (1.9, 3.63)	2.55 (1.9, 3.63)	2.19 (1.72, 2.73)	0.001
Immunoglobulin E, IU/mL	50.6 (18.3, 145.8)	67.1 (31.4, 188)	37 (18.3, 108)	0.007
Immunoglobulin G, g/L	12.1 (10.43, 14.88)	12.5 (10.7, 15.9)	11.8 (10.1, 14.6)	0.067
Immunoglobulin M, g/L	0.94 (0.68, 1.22)	0.95 (0.7, 1.21)	0.94 (0.67, 1.22)	0.998
Cellular immunity ^e^				
CD16+56, %	13.58 (7.58, 22.56)	19.2 (10.3, 29.3)	11.6 (6.43, 17.03)	<0.001
CD16+56 counts, No./*μ*L	115 (69, 176)	97 (41.5, 168.5)	119.5 (77.5, 180.3)	0.012
CD19, %	14.95 (11.46, 21.87)	19.1 (11.7, 27.9)	14.3 (11.4, 19.2)	0.003
CD19 counts, No./*μ*L	131 (82, 191)	141 (87.5, 177)	125.5 (78, 205.25)	0.859
CD3, %	65.0 (53.3, 72.6)	65.16 (51.78, 73.01)	64.83 (53.65,72.44)	0.914
CD3 counts No./*μ*L	574 (294, 873)	272 (185.5, 405)	781 (582.3, 1023.8)	<0.001
CD4, %	39.0 (31.4, 45.5)	32.1 (26.7, 41.5)	41.8 (36.3, 46.8)	<0.001
CD4 counts, No./*μ*L	357 (177, 547)	168 (106.5, 269)	486 (357.8, 651.5)	<0.001
CD8,%	22.0 (14.9, 28.3)	16.0 (10.9, 24.4)	24.0 (18.8, 29.6)	<0.001
CD8 counts, No./*μ*L	182 (88, 319.5)	73 (46.5, 131.5)	263 (166.8, 396)	<0.001
CD4/CD8	1.78 (1.3, 2.52)	1.85 (1.28, 3.11)	1.74 (1.34, 2.29)	0.187

The total number of patients with available data: ^a^*n* (death) = 107, *n* (survival) = 158; ^b^*n* (death) = 107, *n* (survival) = 154; ^c^*n* (death) = 109, *n* (survival) = 128; ^d^*n* (death) = 85, *n* (survival) = 133; ^e^*n* (death) = 87, *n* (survival) = 140.

**Table 4:** Initial pulmonary CT findings of COVID-19 patients.

Characteristics of lung CT No. (%)	All (*n* = 197)	Nonsurviving (*n* = 67)	Surviving (*n* = 130)	*P* value
Pneumonia	180 (91.4%)	63 (94.0%)	117 (90.0%)	0.340
Unilateral lung	29 (14.7%)	12 (17.9%)	27 (20.7%)	0.633
Bilateral lung	148 (75.1%)	53 (79.1%)	95 (73.1%)	0.354
Ground-glass opacity	144 (73.1%)	47 (70.1%)	97 (74.6%)	0.503
Paving stone/reticular/linear	45 (22.8%)	16 (23.9%)	29 (22.3%)	0.803
Consolidation shadow	20 (10.2%)	4 (6.0%)	16 (12.3%)	0.163
Air bronchogram	20 (10.2%)	8 (11.9%)	12 (9.2%)	0.551

**Table 5:** Complications and treatments of COVID-19 patients.

	All (*n* = 293)	Nonsurviving (*n* = 116)	Surviving (*n* = 177)	*P* value
Complications, No. (%)	91 (31.1%)	79 (68.1%)	12 (6.8%)	<0.001
Shock	56 (19.1%)	51 (44.0%)	5 (2.8%)	<0.001
Acute cardiac injury	72 (24.6%)	67 (57.8%)	5 (2.8%)	< 0.001
Acute renal injury	21 (7.2%)	20 (17.2%)	1 (0.6%)	< 0.001
Acute liver injury	19 (6.5%)	12 (10.3%)	7 (4.0%)	0.030
Only one complication	47 (16.0%)	37 (31.9%)	10 (5.6%)	< 0.001
≥2 complications	44 (15%)	42 (36.2%)	2 (1.1%)	< 0.001
Admission to ICU, No. (%)	42 (14.3%)	37 (31.9%)	5 (2.8%)	< 0.001
Oxygen therapy, No. (%)	172 (58.7%)	89 (76.7%)	83 (46.9%)	< 0.001
Nasal catheter oxygen inhalation	152 (51.9%)	72 (62.1%)	80 (45.2%)	0.005
Mask oxygen inhalation	92 (31.4%)	64 (55.2%)	28 (15.8%)	< 0.001
HFBHTI	22 (7.5%)	20 (17.2%)	2 (1.1%)	< 0.001
Noninvasive mechanical ventilation	65 (22.2%)	60 (51.7%)	5 (2.8%)	< 0.001
Invasive mechanical ventilation	22 (7.5%)	22 (19%)	0 (0)	< 0.001
Medical treatment, No. (%)				
Antiviral treatment	247 (84.3%)	87 (75%)	160 (90.4%)	< 0.001
Antibiotic treatment	218 (74.4%)	89 (76.7%)	129 (72.9%)	0.461
Glucocorticoids	139 (47.4%)	79 (68.1%)	60 (33.9%)	< 0.001
Immunoglobulin therapy	142 (48.5%)	71 (61.2%)	71 (40.1%)	< 0.001
Antifungal treatment	9 (3.1%)	8 (6.9%)	1 (0.6%)	0.002
Special treatment, No. (%)				
CRRT	7 (2.4%)	7 (6.0%)	0 (0)	< 0.001
ECMO	2 (0.7%)	2 (1.7%)	0 (0)	0.080
ALSS	2 (0.7%)	2 (1.7%)	0 (0)	0.080

Abbreviations: ICU: intensive care unit; HFBHTI: high-flow breathing humidification therapy instrument; CRRT: continuous renal replacement therapy; ECMO: extracorporeal membrane oxygenation; ALSS: artificial liver support system.

## Data Availability

Data and material related to this manuscript are available from the corresponding authors upon reasonable request.
